# Crystal structure of the leucine-rich repeat ectodomain of the plant immune receptor kinase SOBIR1

**DOI:** 10.1107/S2059798319005291

**Published:** 2019-04-29

**Authors:** Ulrich Hohmann, Michael Hothorn

**Affiliations:** a Structural Plant Biology Laboratory, Department of Botany and Plant Biology, University of Geneva, 1211 Geneva, Switzerland

**Keywords:** leucine-rich repeat, ectodomain, receptor kinase, plant immune signalling, *Arabidopsis*, cell signalling, membrane receptor

## Abstract

Plant-unique membrane receptor kinases with leucine-rich repeat (LRR) extracellular domains are key regulators of development and immune responses. Here, the 1.55 Å resolution crystal structure of the immune receptor kinase SOBIR1 from *Arabidopsis* is presented. The ectodomain structure reveals the presence of five LRRs sandwiched between noncanonical capping domains. The disulfide-bond-stabilized N-terminal cap harbours an unusual β-hairpin structure. The C-terminal cap features a highly positively charged linear motif which was found to be largely disordered in this structure. Size-exclusion chromatography and right-angle light-scattering experiments suggest that SOBIR1 is a monomer in solution. The protruding β-hairpin, a set of highly conserved basic residues at the inner surface of the SOBIR LRR domain and the presence of a genetic missense allele in LRR2 together suggest that the SOBIR1 ectodomain may mediate protein–protein interaction in plant immune signalling.

## Abstract 2

The ectodomain structure of a novel plant membrane receptor kinase with unusual capping domains is reported.

## 1. Introduction   

**Figure 1:**
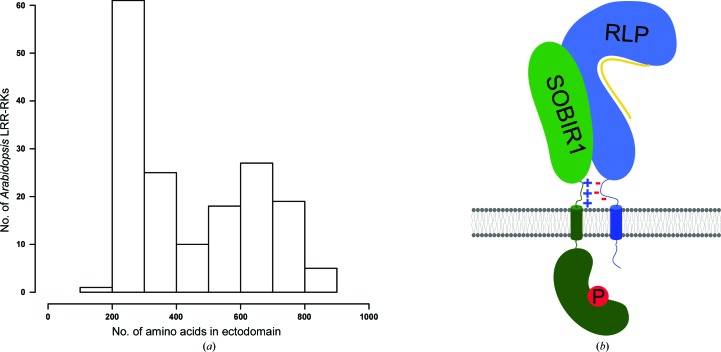
Distribution of leucine-rich repeat receptor kinases in *Arabidopsis*. (*a*) Histogram showing the distribution of *Arabidopsis* LRR-RKs by ectodomain size. The number of residues in the ectodomain is plotted versus the number of LRR-RKs found in the current reference proteome of *A. thaliana*. (*b*) Cartoon model of a putative SOBIR1–receptor-like protein (RLP)–ligand (yellow) complex at the plasma membrane (shown in grey). SOBIR1 kinase domain (green, kidney-shaped) phosphorylation is indicated with a P. Note the presence of charged stretches next to the transmembrane helices (cylinders) in SOBIR1 (positively charged, +) and different RLPs (negatively charged, −).

Plants have evolved a unique set of membrane receptor kinases (LRR-RKs) that are composed of a leucine-rich repeat ectodomain, a transmembrane helix and a dual-specificity kinase domain in the cytoplasm (Shiu & Bleecker, 2001 ▸). The ectodomains of LRR-RKs show a bimodal size distribution (Fig. 1 ▸ *a*). Family members with large ectodomains (15–30 LRRs) represent ligand-binding receptors (Hohmann *et al.*, 2017 ▸). In contrast, SOMATIC EMBRYOGENESIS RECEPTOR KINASEs (SERKs; Schmidt *et al.*, 1997 ▸) with short ectodomains (five LRRs) have been characterized as essential co-receptors (Brandt & Hothorn, 2016 ▸). Ligand binding to large LRR-RKs promotes their association with shape-complementary SERKs at the cell surface, which in turn enables their cytoplasmic kinase domains to interact and to transphosphorylate each other (Santiago *et al.*, 2013 ▸, 2016 ▸; Hohmann, Santiago *et al.*, 2018 ▸). SERKs represent only five of the ∼60 small LRR-RKs in *Arabidopsis* (Fig. 1 ▸ *a*; Dufayard *et al.*, 2017 ▸), but genetic evidence suggests that sequence-related NIK/CIK/CLERK proteins may fulfil similar functions (Hu *et al.*, 2018 ▸; Cui *et al.*, 2018 ▸; Anne *et al.*, 2018 ▸).

Recently, the BIR family of receptor pseudokinases (with five LRRs in the ectodomain) have been defined as negative regulators of SERK co-receptors (Ma *et al.*, 2017 ▸; Hohmann, Nicolet *et al.*, 2018 ▸). Ligand-independent interaction of a BIR and a SERK ectodomain keeps the LRR-RK co-receptor in a basal, inhibited state (Hohmann, Nicolet *et al.*, 2018 ▸). In addition, the structure of POLLEN RECEPTOR-LIKE KINASE 6 (six LRRs in the ectodomain) in complex with a peptide hormone ligand has been reported, but it is presently unclear whether PRK6 represents the receptor or a co-receptor for these peptides (Zhang *et al.*, 2017 ▸).

Here, we report the structure of the functionally distinct plant receptor kinase SOBIR1, which is predicted to have four or five LRRs in its ectodomain (Gao *et al.*, 2009 ▸; Bi *et al.*, 2016 ▸). SOBIR1 was initially found in a suppressor screen of the *bir1-1* mutant, which displays autoimmune phenotypes (Gao *et al.*, 2009 ▸). SOBIR1 loss of function restored wild-type-like growth in *bir1-1*, suggesting that SOBIR1 functions in plant immune signalling (Gao *et al.*, 2009 ▸). Subsequently, it was found that SOBIR1 interacts with receptor-like proteins (RLPs; Liebrand *et al.*, 2013 ▸), a family of plant membrane proteins (∼57 family members in *Arabidopsis*) that harbour LRR ectodomains and a transmembrane helix but lack a cytoplasmic kinase domain (Wang *et al.*, 2008 ▸; Gust & Felix, 2014 ▸; Fig. 1 ▸ *b*). Many RLPs are plant immune receptors that recognize various microbe-associated molecular patterns and their signalling function depends on SOBIR1 (Liebrand *et al.*, 2013 ▸; Zhang *et al.*, 2013 ▸; Jehle *et al.*, 2013 ▸; Albert *et al.*, 2015 ▸; Postma *et al.*, 2016 ▸; Catanzariti *et al.*, 2017 ▸; Wang *et al.*, 2018 ▸; Domazakis *et al.*, 2018 ▸). How different RLPs interact with SOBIR1 to activate plant immune signalling is poorly understood at the molecular level. Presently, it is known that a GxxxG motif in the SOBIR1 transmembrane helix is required for the interaction with different RLPs (Bi *et al.*, 2016 ▸). It has also been demonstrated that the kinase activity of SOBIR1 is essential for its signalling function (van der Burgh *et al.*, 2019 ▸). Here, we present the crystal structure of the SOBIR1 ectodomain from *A. thaliana* (AtSOBIR1) and discuss its implications for plant immune signalling.

## 2. Materials and methods   

### 2.1. Analysis of LRR ectodomain size distribution   

166 *A. thaliana* proteins containing predicted N-terminal LRR and C-terminal kinase domains connected via a single transmembrane helix were identified in Araport11 (https://www.arabidopsis.org/). The LRR ectodomain sequences were isolated by defining putative signal peptides using *SignalP* v.5.0 (http://www.cbs.dtu.dk/services/SignalP/; Almagro Armenteros *et al.*, 2019 ▸) and transmembrane helices using *TMHMM* v.2.0 (http://www.cbs.dtu.dk/services/TMHMM/; Möller *et al.*, 2001 ▸). Data were plotted in *R* (R Core Team, 2014 ▸; Fig. 1 ▸ *a*).

### 2.2. Protein expression and purification   

The coding sequences of AtSOBIR1 (residues 1–270 and 1–183) as well as AtRLP23^1–849^ and AtRLP32^1–818^ were amplified from *A. thaliana* genomic DNA, GP67-RLP23^23–847^ codon-optimized for expression in *Spodoptera frugiperda* was obtained from T. Nürnberger, and PpNLP20-stop codon-optimized for expression in *S. frugiperda* was obtained as a synthetic gene (Twist Bioscience, San Francisco, USA). All protein-coding sequences were cloned into a modified pFastBac vector (Geneva Biotech), which provides a Tobacco etch virus protease (TEV)-cleavable C-terminal StrepII-9×His tag. Shortened expression constructs and signal peptide swaps were constructed using Gibson-assembly cloning strategies (Gibson *et al.*, 2009 ▸).

For protein expression, *Trichoplusia ni* (strain Tnao38; Hashimoto *et al.*, 2010 ▸) cells were infected with 15 ml of virus in 250 ml of cells at a density of 2.3 × 10^6^ cells ml^−1^ and incubated for 26 h at 28°C and 110 rev min^−1^ and then for a further 48 h at 22°C and 110 rev min^−1^. For co-expression, the cells were infected with 10 ml of each virus. Subsequently, the secreted ectodomains were purified from the supernatant by sequential Ni^2+^ (HisTrap Excel; GE Healthcare; equilibrated in 25 m*M* potassium phosphate pH 7.8, 500 m*M* NaCl) and StrepII (Strep-Tactin XT, IBA; equilibrated in 25 m*M* Tris pH 8.0, 250 m*M* NaCl, 1 m*M* EDTA) affinity chromatography. The proteins were further purified by size-exclusion chromatography (on either a Superdex 200 Increase 10/300 GL or a HiLoad 16/600 Superdex 200 pg column, both from GE Healthcare) equilibrated in 20 m*M* sodium citrate pH 5.0, 150 m*M* NaCl. Purified proteins were then concentrated using Amicon Ultra concentrators (molecular-weight cutoff 10 000; Millipore) and purity and structural integrity were assessed by SDS–PAGE and right-angle light scattering (RALS). The molecular weights of the proteins (as determined by RALS) are ∼30.2 kDa for AtSOBIR1^1–270^ and ∼38.1 kDa for AtSOBIR1^1–283^.

### 2.3. Crystallization and crystallographic data collection   

Hexagonal SOBIR1 crystals (∼400 × 80 × 80 µm) developed in hanging drops composed of 1 µl protein solution (20 mg ml^−1^ in 20 m*M* sodium citrate pH 5.0, 150 m*M* NaCl) and 1 µl crystallization buffer [25%(*w*/*v*) PEG 3350, 1 *M* LiCl, 0.1 *M* sodium acetate pH 5.5] suspended in 1 ml of the latter as a reservoir solution. Crystals were cryoprotected by serial transfer into crystallization buffer supplemented with glycerol to a final concentration of 15%(*v*/*v*) and snap-cooled in liquid N_2_.

Native (λ = 1.033 Å, one 360° wedge at 0.1° oscillation) and redundant sulfur single-wavelength anomalous dispersion (SAD) data (λ = 2.079 Å, three 360° wedges at 0.1° oscillation, with χ set to −10°, 0° and 10°) were collected to 1.75 and 3.12 Å resolution, respectively, on beamline X06DA at the Swiss Light Source (SLS), Villigen, Switzerland equipped with a PILATUS 2M-F detector (Dectris) and a multi-axis gonio­meter. A second native data set to 1.55 Å resolution was recorded from a different crystal. Data processing and scaling were performed with *XDS* and *XSCALE*, respectively (version January 2018; Kabsch, 2010 ▸).

### 2.4. Structure determination and refinement   

The anomalous signal in the scaled SAD data set extended to ∼4.0 Å resolution when analysed with *phenix.xtriage* (Zwart *et al.*, 2005 ▸; Adams *et al.*, 2010 ▸). The structure was solved using the molecular replacement/single-wavelength anomalous dispersion (MR-SAD) method as implemented in *Phaser* (McCoy *et al.*, 2007 ▸). An alignment of the SOBIR1 and SERK1 ectodomains (which share ∼30% sequence identity) was prepared in *HHpred* (Zimmermann *et al.*, 2018 ▸) and input into *CHAINSAW* (Stein, 2008 ▸). The LRR ectodomain of SERK1 (PDB entry 4lsc; Santiago *et al.*, 2013 ▸) with non-identical side chains trimmed to alanines was used as a search model. *Phaser* returned a single solution in space group *P*6_5_, comprising a dimer in the asymmetric unit. The first molecule had a *Phaser* rotation-function *Z*-score (RFZ) of 3.5, a translation-function *Z*-score (TFZ) of 6.6 and an associated log-likelihood gain (LLG) of 60. The TFZ for the second molecule was 14.4, with a final refined LLG of 351. The resulting partial model [the starting figure of merit (FOM) was 0.355 at 3.12 Å resolution] was used to locate nine putative sulfur sites by log-likelihood-gradient completion in *Phaser* (the final FOM was 0.428). Density-modification and phase extension to 1.75 Å resolution in *phenix.resolve* (Terwilliger, 2003 ▸) yielded a readily interpretable electron-density map. The structure was completed by alternating cycles of manual model building and correction in *Coot* (Emsley & Cowtan, 2004 ▸) and restrained TLS refinement against a 1.55 Å resolution native data set in *phenix.refine* (Afonine *et al.*, 2012 ▸; Table 1 ▸). Inspection of the final model with *phenix.molprobity* (Chen *et al.*, 2010 ▸) revealed excellent stereochemistry (Table 1 ▸). Structural representations were generated in *PyMOL* (http://pymol.org) and *UCSF Chimera* (Pettersen *et al.*, 2004 ▸). Electrostatic potentials were calculated using the *PyMOL* *APBS* plugin (Jurrus *et al.*, 2018 ▸). To visualize phased anomalous difference maps, |*F*_A_| values and phase shifts were calculated from the SAD data set in *XPREP* (Bruker) and input into *ANODE* (Thorn & Sheldrick, 2011 ▸) together with the final SOBIR1 coordinate file. The resulting map file was converted to CCP4 format using *SHELX*2*MAP*. The crystallographic coordinates and structure factors have been deposited in the Protein Data Bank (http://rcsb.org) as entry 6r1h. Native and sulfur SAD diffraction images and *XDS* processing files have been deposited at zenodo.org (https://doi.org/10.5281/zenodo.2594485 and https://doi.org/10.5281/zenodo.2595891, respectively).

### 2.5. Analytical size-exclusion chromatography   

Analytical size-exclusion chromatography (SEC) experiments were performed on a Superdex 200 Increase 10/300 GL column (GE Healthcare) pre-equilibrated in 20 m*M* sodium citrate pH 5.0, 250 m*M* NaCl. 200 µg of protein, injected in a volume of 100 µl, was loaded onto the column and elution at 0.75 ml min^−1^ was monitored by ultraviolet absorbance at λ = 280 nm. Peak fractions were analysed by SDS–PAGE.

### 2.6. Right-angle light scattering   

The oligomeric state of SOBIR1 was analysed by size-exclusion chromatography paired with a right-angle light-scattering (RALS) and a refractive-index (RI) detector using an OMNISEC RESOLVE/REVEAL combined system. Calibration of the instrument was carried out using a BSA standard (Thermo Scientific Albumin Standard). 100 µg of protein in a volume of 50 µl was separated on a Superdex 200 Increase column (GE Healthcare) in 20 m*M* sodium citrate pH 5.0, 250 m*M* NaCl at a column temperature of 35°C and a flow rate of 0.7 ml min^−1^. The data were analysed using the *OMNISEC* software (v.10.41).

## 3. Results   

**Figure 2:**
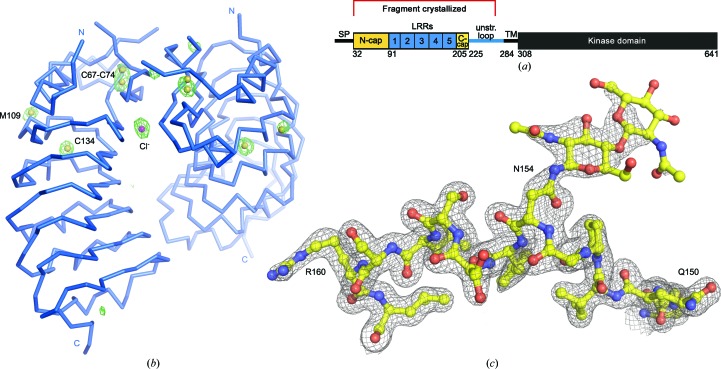
Structure solution of SOBIR1. (*a*) Schematic representation of SOBIR1 (SP, signal peptide; TM, transmembrane helix; unstr. loop, unstructured loop; N-­cap/C-cap, N/C-terminal capping domain). The fragment crystallized is indicated in red. (*b*) C^α^ trace of the SOBIR1 crystallographic dimer (in blue) including a phased anomalous difference map contoured at 5.0σ (green mesh), the eight S atoms (yellow spheres) and a putative chloride anion (magenta). (*c*) Example region of the SOBIR1 structure, including the N-glycosylated Asn154 (yellow ball-and-stick representation), with the final (2*F*_o_ − *F*_c_) map contoured at 1.2σ.

We obtained the *A. thaliana* SOBIR1 ectodomain (residues 1–270) by secreted expression in insect cells (Fig. 2 ▸ *a*; see Section 2). The N-glycosylated protein was crystallized using the vapour-diffusion method and the structure was solved by MR-SAD on beamline X06DA at the Swiss Light Source (see Section 2 and Table 1 ▸; Basu *et al.*, 2019 ▸). The solution in space group *P*6_5_ comprises a dimer in the asymmetric unit, with the nine putative sulfur sites corresponding to a disulfide bridge in the N-terminal LRR capping domain, to a free cysteine and a methionine residue in the LRR core and to a free ion, which we interpreted as a chlorine anion originating from the crystallization buffer (Fig. 2 ▸ *b*). The model was refined against an isomorphous, high-resolution native data set at 1.55 Å resolution. An example region of the final (2*F*_o_ − *F*_c_) map is shown in Fig. 2 ▸(*c*), highlighting the only N-glycan located in the structure, which was attached to Asn154.

**Figure 3:**
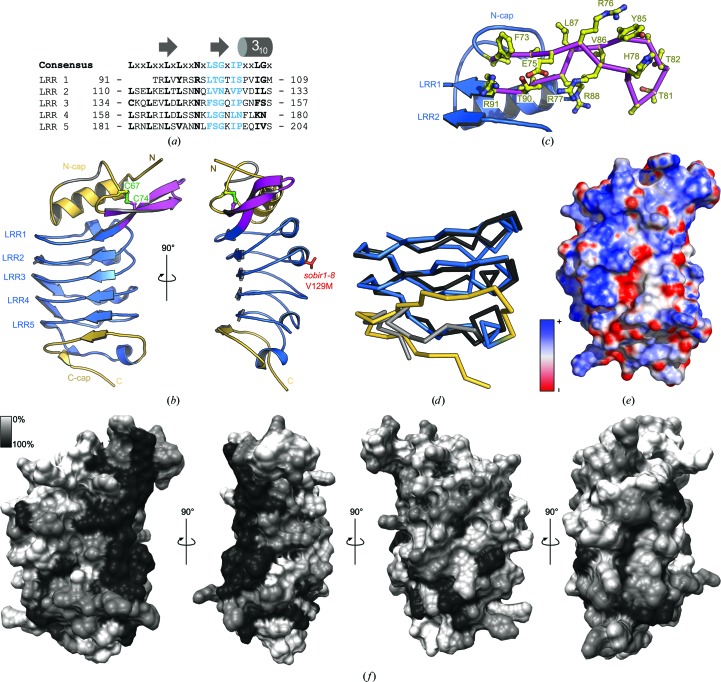
The SOBIR1 ectodomain harbours five LRRs and unusual capping domains. (*a*) Sequence alignment of the five SOBIR1 LRRs, with the canonical consensus sequence in black and the plant-specific LRR motif in blue. The LRR consensus sequence and a secondary-structure assignment calculated with *DSSP* (Kabsch & Sander, 1983 ▸) are shown alongside. (*b*) Front (left) and *y*-axis-rotated side (right) views of the SOBIR1 LRR domain (blue ribbon diagram), with N- and C-terminal capping domains shown in yellow, the SOBIR1-specific extended β-hairpin in magenta and a disordered loop in the N-­terminal capping domain (residues 57–63) in grey. The disulfide bond (in green) and Val129 (in red), which is mutated to Met in *sobir1-8*, are shown in bond representations. (*c*) Close-up view of the extended β-hairpin (magenta C^α^ trace, with side chains shown in ball-and-stick representation in yellow) in the SOBIR1 ectodomain (blue ribbon diagram). (*d*) C^α^ traces of a structural superposition of the ectodomains of SOBIR1 (blue, C-terminal capping domain in yellow) and SERK3 (PDB entry 4mn8; Sun, Li *et al.*, 2013 ▸; black, C-terminal capping domain in grey). (*e*) Surface representation of the SOBIR1 ectodomain coloured according to the electrostatic surface potential (blue, negative; red, positive). (*f*) Surface representation of the SOBIR1 ectodomain coloured according to SOBIR1 sequence conservation, comparing SOBIR orthologues from different plant species (sequences are shown in Fig. 4 ▸ *c*). Note the presence of a highly conserved patch at the outer edge of the inner surface, ranging from the N-terminal cap through LRRs 1–5.

The refined model reveals the presence of five LRRs in the SOBIR1 ectodomain, not four as initially proposed (Gao *et al.*, 2009 ▸; Figs. 3 ▸ *a* and 3 ▸ *b*). A genetic missense allele (*sobir1-8*; Val129 to Met), which causes a weak *sobir1* loss-of-function phenotype, maps to the outer face of the LRR core in LRR2 (Gao *et al.*, 2009 ▸). The SOBIR1 LRR core is masked by an N-terminal capping domain, as found in many plant LRR-RKs (residues 34–90, shown in yellow in Fig. 3 ▸ *b*; Hohmann *et al.*, 2017 ▸). Loop residues 57–63 appear disordered in our structure (shown in grey in Fig. 3 ▸ *b*). The N-terminal cap features a protruding, unusual β-hairpin structure (shown in magenta in Fig. 3 ▸ *b*), which presents several conserved basic and hydrophobic amino acids on its surface (Fig. 3 ▸ *c*).

**Figure 4:**
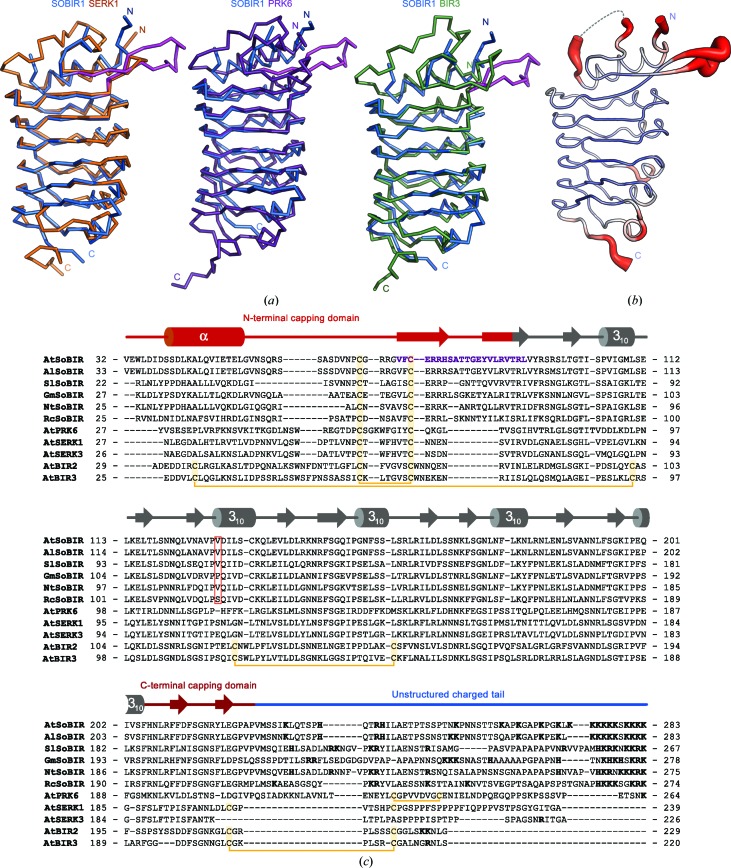
AtSOBIR1 shares a common architecture with other small plant LRR-RKs. (*a*) C^α^ traces of structural superposition of the ectodomain of SOBIR1 (blue) with AtSERK1 (left, orange; PDB entry 4lsc; Santiago *et al.*, 2013 ▸), AtPRK6 (centre, purple; PDB entry 5yah; Zhang *et al.*, 2017 ▸) and AtBIR3 (right, green; PDB entry 6fg8; Hohmann, Nicolet *et al.*, 2018 ▸). The SOBIR1-unique extended β-hairpin is highlighted in magenta. (*b*) Ribbon diagram of the SOBIR1 ectodomain with C^α^ atoms coloured according to their crystallographic temperature factors (red, high; blue, low). Residues 57–63, which are missing in the structure, are indicated by a dotted line. The N- and C-termini as well as an N-terminal capping-domain loop and the extending β-hairpin appear to be flexible, in contrast to the rigid and well ordered LRR core. (*c*) Structure-based sequence alignment of the ectodomains of SOBIR1 from *A. thaliana* [UniProt (http://www.uniprot.org) identifier Q9SKB2], *A. lyrata* (UniProt identifier D7LEA5), *Solanum lycopersicum* (UniProt identifier K4C8Q3), *Glycine max* (UniProt identifier I1JXE0), *Nicotiana tabacum* (UniProt identifier Q8LP72) and *Ricinus communis* (UniProt identifier B9RAQ8) as well as *A. thaliana* SERK1 (UniProt identifier Q94AG2), SERK3 (UniProt identifier Q94F62), BIR2 (UniProt identifier Q9LSI9) and BIR3 (UniProt identifier O04567). Shown alongside is a secondary-structure assignment (calculated with *DSSP*; Kabsch & Sander, 1983 ▸), with the N- and C-terminal capping domains highlighted in red and a unstructured region at the C-terminus shown in blue. Disulfide bridges are shown in yellow and the SOBIR1-specific β-hairpin in purple; the position of Val129 of AtSOBIR1, which is mutated to methionine in *sobir1-8*, is indicated by a red box; positively charged residues in the unstructured C-terminal tail are highlighted in bold.

A highly basic, low-complexity region is located at the C-termini of SOBIR ectodomains from different plant species (Fig. 4 ▸ *c*). In line with this, the C-terminal capping domain, which in plant LRR-RKs is normally terminated by a well defined disulfide bond (Hohmann *et al.*, 2017 ▸), is found to be largely disordered in our SOBIR1 structure (Fig. 3 ▸ *b*). This is reminiscent of the LRR-RK SERK3, which contains a proline-rich sequence at the C-terminus of its ectodomain and the C-terminal capping domain of which was also found to be largely unstructured in different SERK3–LRR-RK complex structures (Fig. 3 ▸ *d*; Sun, Li *et al.*, 2013 ▸; Sun, Han *et al.*, 2013 ▸).

Analysis of the electrostatic surface potential of the SOBIR1 ectodomain revealed several basic patches on the inner side of the LRR solenoid (Fig. 3 ▸ *e*), some of which are highly conserved among SOBIR orthologues from different plant species (Figs. 3 ▸ *f* and 4 ▸ *c*).

We next compared the SOBIR1 ectodomain with other plant LRR-RKs. A structural homology search with *DALI* (Holm & Sander, 1993 ▸) returned several large and small LRR ectodomains as the top hits. We focused our analysis on plant LRR-RKs with small ectodomains. The ectodomain of the SERK1 co-receptor kinase (PDB entry 4lsc; Santiago *et al.*, 2013 ▸) has a *DALI Z*-score of 21.6 and superimposes with SOBIR1 with a root-mean-square deviation (r.m.s.d.) of ∼1 Å comparing 123 corresponding C^α^ atoms (shown in yellow in Fig. 4 ▸ *a*). SERK1 shares the number of LRRs and the N-terminal capping domain with SOBIR1, but has a canonical, disulfide-bond-stabilized C-terminal cap (Hohmann *et al.*, 2017 ▸). The peptide-ligand-sensing PRK6 ectodomain (PDB entry 5yah; *DALI Z*-score 19.2; Zhang *et al.*, 2017 ▸) superimposes with an r.m.s.d. of ∼1.2 Å comparing 99 corresponding C^α^ atoms (shown in purple in Fig. 4 ▸ *a*). The ectodomain of the BIR3 LRR receptor pseudokinase (PDB entry 6fg8; *DALI Z*-score 20.7; Hohmann, Nicolet *et al.*, 2018 ▸) aligns with an r.m.s.d. of ∼1.2 Å comparing 120 corresponding C^α^ atoms (shown in green in Fig. 4 ▸ *a*). Together, our structural comparisons reveal that functionally diverse plant receptor ectodomains share strong structural homology, with the exception of the N-terminal and C-terminal capping domains. In SOBIR1, a unique β-hairpin protrudes from the N-terminal cap (Figs. 3 ▸ *b* and 4 ▸ *a*). This hairpin and a loop structure connecting the N-terminal capping helix to the β-hairpin both appear to be flexible, as judged by analysis of the crystallographic temperature factors (Fig. 4 ▸ *b*).

**Figure 5:**
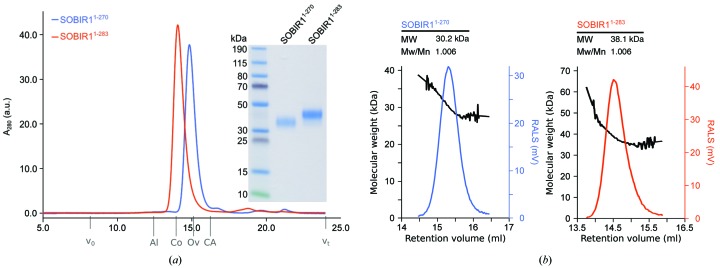
The AtSOBIR1 ectodomain is a monomer in solution. (*a*) Analytical size-exclusion chromatography traces of SOBIR1^1–270^ (blue) and SOBIR1^1–283^ (orange) with the SDS–PAGE analysis of pooled peak fractions alongside. The void volume (*v*_0_), the total column volume (*v*_t_) and the elution volumes for molecular-mass standards (Al, aldolase, 158 kDa; Co, conalbumin, 75 kDa; Ov, ovalbumin, 43 kDa; CA, carbonic anhydrase, 29 kDa) are indicated. (*b*) Analysis of the oligomeric state of AtSOBIR1. Raw right-angle light-scattering traces (blue and orange) and extrapolated molecular weights (black) of SOBIR1^1–270^ and SOBIR1^1–283^ are shown, with a summary table including the observed molecular weight (MW) and the dispersity (Mw/Mn) alongside. The theoretical molecular weights are 27.4 kDa for SOBIR1^1–270^ and 33.3 kDa for SOBIR1^1–283^.

We located a crystallographic SOBIR1 dimer in our crystals, which would bring the C-terminal capping domains (connecting to the transmembrane helices in the context of the full-length receptor) into close proximity (Fig. 2 ▸ *a*). Analysis of the crystal packing with the *PISA* server (Krissinel & Henrick, 2007 ▸) revealed a rather small complex interface of ∼1000 Å^2^ formed by several salt bridges, hydrogen bonds and a few hydrophobic contacts. We next performed analytical size-exclusion chromatography and right-angle light-scattering experiments to assess the oligomeric state of the SOBIR1 extracellular domain in solution. At pH 5.0 (which corresponds to the pH associated with the plant cell-wall compartment), we found our SOBIR1^1–270^ construct used for crystallization to be a monodisperse monomer with an approximate molecular weight of 30.2 kDa (the calculated molecular weight is 27.4 kDa; Figs. 5 ▸ *a* and 5 ▸ *b*). A longer construct that includes the complete extracellular region of SOBIR1 up to the transmembrane helix (residues 1–283) also behaves as a monomer, with an observed molecular weight of 38.1 kDa (the calculated molecular weight is 33.3 kDa; Figs. 5 ▸ *a* and 5 ▸ *b*). These experiments in solution suggest that the SOBIR1 dimer observed in our structure is likely to represent a crystal-packing artefact.

**Figure 6:**
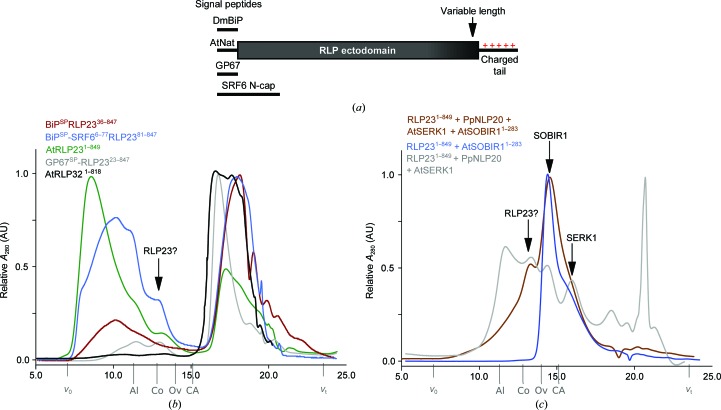
Expression and purification of AtRLPs. (*a*) Schematic representation of different RLP expression constructs. Variations include different signal peptides (DmBIP, signal peptide from *D. melanogaster* binding protein; AtNat, native *A. thaliana* signal peptide, GP67, baculoviral glycoprotein 67 signal peptide, SRF6 N-cap, utilization of the whole SRF6 N-terminal capping domain) and variable construct lengths (including or omitting the charged C-terminal tail). (*b*) Example analytical size-exclusion chromatography traces from RLP purifications. A peak for a monomeric, neither aggregated nor degraded RLP ectodomain (MW of ∼100 kDa) would be expected at an elution volume of ∼13 ml (indicated by an arrow). The void volume (*v*_0_), the total column volume (*v*_t_) and the elution volumes for molecular-mass standards (Al, aldolase, 158 kDa; Co, conalbumin, 75 kDa; Ov, ovalbumin, 43 kDa; CA, carbonic anhydrase, 29 kDa) are indicated. (*c*) Example analytical size-exclusion chromatography traces for AtRLP23 purifications from co-expression with SOBIR^1–283^ alone (blue), with the RLP23 ligand PpNLP20 and the co-receptor AtSERK1 (grey), and with SOBIR1, SERK1 and the ligand PpNLP20 (brown). Labels are as in (*b*).

We next sought to test the genetic and *in vivo* biochemical finding that SOBIR1 forms heteromeric complexes with RLPs. To this end, we produced the LRR ectodomains of RLP23 and RLP32 from *A. thaliana* by secreted expression in insect cells (see Section 2). Utilization of the native signal peptide (AtNat), the baculoviral glycoprotein 67 signal peptide (GP67) or the signal peptide from *Drosophila melanogaster* binding protein (DmBiP; Fig. 6 ▸ *a*) all lead to accumulation and secretion of RLP23, but we found that the protein either aggregated or degraded in analytical size-exclusion chromatography assays (Fig. 6 ▸ *b*). RLP32 showed a similar behaviour when expressed using the different signal peptides (Fig. 6 ▸ *b*). We next varied the C-terminus of these constructs, omitting the positively charged C-terminal tail (Fig. 6 ▸ *a*). However, this did not improve the behaviour of the resulting recombinant proteins. We next replaced the flexible N-terminal capping domain of RLP23 with the N-terminal cap of the STRUBBELIG RECEPTOR FAMILY 6 LRR-RK, but the resulting chimeric protein was still rapidly degraded in our preparations. Finally, we co-expressed the RLP23^1–849^ ectodomain with its bona fide peptide ligand NLP20 (Böhm *et al.*, 2014 ▸), the SOBIR1^1–283^ ectodomain and the LRR domain of the putative SERK1 co-receptor kinase (Albert *et al.*, 2015 ▸). However, co-expression of the ectodomains of the putative signalling complex also did not improve the biochemical behaviour of RLP23, and thus we could not assess the role of the SOBIR1 ectodomain in immune-complex formation.

## 4. Discussion   

The plant membrane receptor kinase SOBIR1 is a central regulator of plant immunity. It is required for signal transduction of conserved microbe-associated molecular patterns sensed by receptor-like proteins which lack cytoplasmic signalling domains (Gust & Felix, 2014 ▸). In genetic terms, the deletion of BIR1 or the overexpression of SERK3 leads to an overactivation of immune signalling (Gao *et al.*, 2009 ▸; Domínguez-Ferreras *et al.*, 2015 ▸). In both cases these effects can be suppressed by the deletion of SOBIR1, suggesting that an RLP–SOBIR1–SERK complex, negatively regulated by BIR1, controls this immune response in wild-type plants. SOBIR1 and RLPs are likely to form heteromeric signalling complexes. The conserved GxxxG motifs in their transmembrane regions, but neither the SOBIR1 LRR ectodomain nor the kinase domain, are required for this interaction to occur *in planta* (Bi *et al.*, 2016 ▸). However, both an active kinase domain and the SOBIR1 LRR ectodomain are required for signalling (Bi *et al.*, 2016 ▸; van der Burgh *et al.*, 2019 ▸). The crystal structure of the SOBIR1 ectodomain reveals five LRRs sandwiched between unusual N-terminal and C-terminal capping domains (Fig. 2 ▸).

The known genetic *sobir1-8* missense allele (Gao *et al.*, 2009 ▸) maps to the surface of LRR2, a unique β-hairpin structure presents conserved aromatic amino acids (Phe73 and Tyr85) at the surface of the domain, and the inner surface of the LRR core contains conserved patches of basic residues. Together, these structural observations argue for a role of the SOBIR1 ectodomain in mediating protein–protein inter­actions at the cell surface. In this respect, it is of note that the area corresponding to the disordered loop region in the SOBIR1 N-terminal cap (shown in grey in Fig. 2 ▸ *b*) is involved in receptor–ligand interactions in the structurally related SERK LRR-RKs (Santiago *et al.*, 2013 ▸, 2016 ▸; Sun, Li *et al.*, 2013 ▸; Hohmann *et al.*, 2017 ▸; Hohmann, Santiago *et al.*, 2018 ▸).

At this point, we can only speculate about the nature of these protein–protein interactions. RLPs involved in plant development have been shown to directly interact with the ectodomains of large ligand-binding LRR-RKs, contributing to the sensing of small protein hormones (Lin *et al.*, 2017 ▸). We speculate that the ectodomain of SOBIR1 may play a similar role in RLP-mediated immune signalling, potentially by contributing conserved interaction surfaces from the LRR core and/or from the protruding β-hairpin, as seen in our artificial crystallographic dimer (Figs. 2 ▸ *b* and 5 ▸). In fact, the rather basic inner surface of the SOBIR1 LRR core (Fig. 3 ▸ *e*) may provide a docking platform for the highly negatively charged sequence stretch in different RLPs located adjacent to the RLP C-terminal capping domain (Gust & Felix, 2014 ▸).

Alternatively, the SOBIR1 ectodomain could represent a binding platform for pathogen- or plant cell-wall-derived ligands, based on the structural and biochemical observation that LRR domains with few repeats such as the plant RPK6 or animal lymphocyte receptors have evolved to bind peptide and small-molecule ligands (Zhang *et al.*, 2017 ▸; Han *et al.*, 2008 ▸).

To test these various hypotheses, we expressed different RLPs from *Arabidopsis* for biochemical interaction studies, as previously reported for the RLP23 ectodomain (Albert *et al.*, 2015 ▸). However, using different expression and purification strategies (different signal peptides, construct lengths and co-expression with a secreted peptide ligand, SOBIR1 and SERKs) we could not obtain well behaving samples of RLP23 or RLP32 for quantitative binding assays. In our hands, it is thus presently not possible to dissect the contribution of the SOBIR1 ectodomain to RLP ligand sensing, complex formation and signalling at the biochemical and structural levels.

## Supplementary Material

Crystal structure of the LRR ectodomain from the plant immune receptor kinase SOBIR1 from Arabidopsis thaliana - native dataset URL: https://doi.org/10.5281/zenodo.2594485

Crystal structure of the LRR ectodomain from the plant immune receptor kinase SOBIR1 from Arabidopsis thaliana - sulfur SAD datasets URL: https://doi.org/10.5281/zenodo.2595891

PDB reference: LRR ectodomain of the receptor kinase SOBIR1 from *Arabidopsis thaliana*, 6r1h

## Figures and Tables

**Table 1:** Crystallographic data-collection, phasing and refinement statistics Values in parentheses are for the highest resolution shell.

	Sulfur MR-SAD	Native 1	Native 2 (high resolution)
Data collection			
Wavelength (Å)	2.079	1.033	1.001
Space group	*P*6_5_	*P*6_5_	*P*6_5_
*a*, *b*, *c* (Å)	81.9, 81.9, 109.8	81.9, 81.9, 109.8	82.3, 82.3, 109.9
α, β, γ (°)	90, 90, 120	90, 90, 120	90, 90, 120
Resolution (Å)	43.41–3.12 (3.18–3.12)	41.43–1.75 (1.80–1.75)	43.5–1.55 (1.65–1.55)
*R*_meas_ †	0.07 (0.23)	0.14 (3.59)	0.07 (2.73)
CC_1/2_ † (%)	100 (100)	100 (64.9)	100 (51.3)
〈*I*/σ(*I*)〉†	47.05 (13.4)	17.6 (1.0)	25.0 (0.9)
Completeness† (%)	100 (100)	100 (99.9)	99.9 (99.3)
Multiplicity†	29.2 (22.6)	21.7 (21.1)	18.5 (12.6)
Wilson *B* factor† (Å^2^)	37.2	38.0	34.1
Phasing			
Resolution (Å)	43.41–3.12		
No. of sites	9		
FOM‡	0.428		
Refinement			
Resolution (Å)			43.5–1.55 (1.58–1.55)
No. of reflections			60266 (2528)
*R*_work_/*R*_free_ §			0.172/0.188 (0.416/0.423)
No. of atoms			
Protein			2949
Glycan			28
Buffer			15
Chloride			1
Water			254
*B* factors§ (Å^2^)			
Protein			40.2
Glycan			85.1
Buffer			69.1
Chloride			32.1
Water			43.1
R.m.s. deviations§			
Bond lengths (Å)			0.005
Bond angles (°)			1.05
*MolProbity* results			
Ramachandran outliers (%)			0
Ramachandran favoured (%)			95.24
*MolProbity* score			1.40
PDB code			6r1h

† As defined in *XDS* (Kabsch, 2010 ▸).

‡ Final figure of merit of the MR-SAD experiment as defined in *Phaser* (McCoy *et al.*, 2007 ▸).

§ As defined in *phenix.refine* (Afonine *et al.*, 2012 ▸).
